# Cost of diabetes mellitus in Africa: a systematic review of existing literature

**DOI:** 10.1186/s12992-017-0318-5

**Published:** 2018-01-16

**Authors:** Chipo Mutyambizi, Milena Pavlova, Lumbwe Chola, Charles Hongoro, Wim Groot

**Affiliations:** 1 Population Health, Health Systems and Innovation, Human Sciences Research Council, HSRC Building, 134 Pretorius Street, Pretoria, 0002 South Africa; 2 Department of Health Services Research; CAPHRI, Maastricht University Medical Centre, Faculty of Health, Medicine and Life Sciences, Maastricht University, Maastricht, The Netherlands; 3 School of Public Health, Faculty of Health Sciences, University of the Witwatersrand, Johannesburg, South Africa

**Keywords:** Diabetes, Africa, Cost of illness, Economic burden, Healthcare costs

## Abstract

**Background:**

There is an increasing recognition that non communicable diseases impose large economic costs on households, societies and nations. However, not much is known about the magnitude of diabetes expenditure in African countries and to the best of our knowledge no systematic assessment of the literature on diabetes costs in Africa has been conducted. The aim of this paper is to capture the evidence on the cost of diabetes in Africa, review the methods used to calculate costs and identify areas for future research.

**Methods:**

A desk search was conducted in Pubmed, Medline, Embase, and Science direct as well as through other databases, namely Google Scholar. The following eligibility criteria were used: peer reviewed English articles published between 2006 and 2016, articles that reported original research findings on the cost of illness in diabetes, and studies that covered at least one African country. Information was extracted using two data extraction sheets and results organized in tables. Costs presented in the studies under review are converted to 2015 international dollars prices (I$).

**Results:**

Twenty six articles are included in this review. Annual national direct costs of diabetes differed between countries and ranged from I$3.5 billion to I$4.5 billion per annum. Indirect costs per patient were generally higher than the direct costs per patient of diabetes. Outpatient costs varied by study design, data source, perspective and healthcare cost categories included in the total costs calculation. The most commonly included healthcare items were drug costs, followed by diagnostic costs, medical supply or disposable costs and consultation costs. In studies that reported both drug costs and total costs, drug costs took a significant portion of the total costs per patient. The highest burden due to the costs associated with diabetes was reported in individuals within the low income group.

**Conclusion:**

Estimation of the costs associated with diabetes is crucial to make progress towards meeting the targets laid out in Sustainable Development Goal 3 set for 2030. The studies included in this review show that the presence of diabetes leads to elevated costs of treatment which further increase in the presence of complications. The cost of drugs generally contributed the most to total direct costs of treatment. Various methods are used in the estimation of diabetes healthcare costs and the costs estimated between countries differ significantly. There is room to improve transparency and make the methodologies used standard in order to allow for cost comparisons across studies.

**Electronic supplementary material:**

The online version of this article (10.1186/s12992-017-0318-5) contains supplementary material, which is available to authorized users.

## Background

Diabetes mellitus is now one of the major challenges for many of the health systems in Africa [1]. For a long time, diabetes was considered a disease of affluence [2] and Africa was considered relatively free from the disease [3]. Epidemiological transition, demographic and nutrition changes have often been cited as the major driving forces in the rapid increase of the number of individuals with diabetes in Africa [1, 4]. In 2015 diabetes was one of the leading causes of non-communicable diseases (NCD) death, contributing 1.5 million deaths globally [5] and 321,100 deaths in the African region [6]. A staggering 79% of these deaths in Africa occurred among people below the age of 60 [6]. The International Diabetes Federation (IDF) estimates that the number of people with diabetes in Africa will increase from 14.2 million in 2015 to 34.2 million in 2040 [6]. More than half of the adults with diabetes in Africa live in some of the region’s most populous countries: South Africa, the Democratic Republic of Congo, Nigeria and Ethiopia [6].

The increase in prevalence and premature mortality due to diabetes imposes huge financial costs to households and governments [7] whist placing immense pressure on the already overstretched healthcare systems in Africa [8]. The urgent need to address the NCD pandemic is now also entrenched in one of the 17 Sustainable Development Goals (SDG) [9]. Amongst other targets, SDG 3 affirms a commitment to ensure a reduction by one third in premature mortality due to NCDs and the achievement of universal health coverage by 2030. The accomplishment of this target will amount to a reduction in NCD prevalence, which will potentially offset the costs associated with NCDs and will contribute to the elimination of inequalities in health care costs.

Individuals with diabetes are likely to experience one or more chronic illnesses such as heart disease, and kidney disease [6]. As the prevalence of diabetes increases, the macro vascular and micro vascular complications associated with the disease will make it a very costly disease to manage, consuming an ever increasing vast amount of resources and national healthcare budgets [10]. Notwithstanding the paucity of data on diabetes in Africa, the IDF estimates that Africa spends 7% of its healthcare budget on diabetes [6]. Healthcare expenditure due to diabetes in 2015 was USD 3.4 billion and is estimated to increase to USD 5.5 billion in 2040 [6]. However, these estimates are uncertain because 66.7% of people with diabetes in Africa are assumed to be undiagnosed [6]. This unmet need for diabetes diagnosis is a result of weak health systems in many African countries that fail to screen patients for diabetes [11]. Efforts to manage the disease in African countries are further hampered by a lack of diabetes education and the role of traditional healers [3]. On a continent in which resources are limited and health finance is heavily reliant on out of pocket (OOP) payments, African governments grapple with the costs of diabetes management [1, 8].

The usefulness of the results presented in cost of illness (COI) studies has often been questioned due to the wide variation in methods used [12, 13]. Despite this COI studies have been conducted across a wide range of diseases and continue to play an important role in conducting full economic evaluations of treatments and other healthcare interventions [12, 14]. An assessment of diabetes costs in African countries is important for various reasons. The prevalence of diabetes is rapidly growing and mostly affects young adults. This has the potential to affect economic productivity and it also threatens the livelihood of many families within the region. The region has a high unmet need for diabetes diagnoses and treatment [11]. It is heavily reliant on OOP healthcare financing and is reported to have the lowest spending on diabetes when compared to other regions [6, 8]. An estimation of the costs associated with diabetes can give insight into the benefits of disease prevention and can facilitate the design and adoption of cost effective treatment options. This is particularly important for countries like South Africa that are looking to achieve universal health coverage.

Recent reviews on costs of diabetes have almost exclusively focused on developed countries, the United States of America and European countries, with a small number of African countries included [15–17]. Reviews that focused on diabetes in Africa did not strictly look into the COI but aimed to investigate the epidemiology of diabetes and its complications as well as the challenges in access to diagnosis and care for diabetes [18–20]. Hall et al. and Kengne et al. build up on these studies on diabetes in Africa by retrieving information on the costs associated with diabetes [1, 21]. A study published in 2007 by Jean Claude Mbanya concludes that the costs associated with diabetes consume a huge proportion of annual health budgets in Sub-Saharan African countries, many of which already run healthcare budget deficits [8]. Although there was limited information on diabetes COI in Sub-Saharan Africa at the time, the author draws from other studies and show that diabetes consumed up to 8.1% of the healthcare budget in Tanzania for the year 1989–1990 and 3.5% in Cameroon for the year 2001–2002 [8]. Our review expands on these previous studies and reviews the costs of diabetes within Africa. The review seeks to: (1) capture the evidence presented in the literature on the overall direct and indirect costs of diabetes in Africa that have been published since 2006; (2) review the methods that have been used to calculate the costs of diabetes in Africa; (3) identify areas for future research.

## Methods

This literature review was guided by the Preferred Reporting Items for Systematic Reviews and Meta-Analysis (PRISMA) guidelines [22]. The guidelines provide a minimum set of reporting items aimed at improving the quality of systematic reviews and meta-analyses. The collection and review of articles was conducted between January and May 2017.

### Literature search strategy

A desk research was conducted in order to identify articles for inclusion. The literature search was undertaken in PubMed, EMBASE, Medline and Science Direct. Searches were also conducted in the WHO Global Health Library and IDF. Additional searches were also undertaken in Google Scholar and manual searches were also undertaken in Google in order to identify publications that may not be indexed in international databases. Our PubMed search strategy is provided in an additional file (see Additional file 1). All searches were conducted in January 2017. Keywords were carefully selected to ensure that all relevant material was included and to also avoid including unnecessary articles. The keywords and search strategy applied was checked by an experienced university librarian. The search terms were adapted for each database using (1) terms for African (Africa OR each country in Africa) AND (2) terms related to diabetes (diabetes OR diabetes mellitus OR diabetic) AND (3) terms denoting costs (expenditure OR cost OR economic burden OR healthcare cost OR cost of illness).

Following the search for articles, the next step involved duplicates removal, this was done using EndNote. In the first level of screening, article titles were screened by one reviewer (CM) to identify studies to include in the review. If based on the article title alone, it was unclear whether the study was relevant with regard to our research question, the article’s abstract was then screened. The third and final step involved assessing for eligibility via full text review to determine if the inclusion and exclusion criteria were satisfied. Articles were then downloaded for a full-text review. In circumstances where the reviewer or a library assistant failed to access the full text, emails were sent to the corresponding authors whose contact details could be obtained, requesting a copy of the missing studies. Reference lists of all eligible articles and reference lists of excluded reviews were also screened for additional relevant material. In every step of the selection process if there was any doubt regarding the inclusion/exclusion of an article another author was consulted.

### Inclusion criteria

Eligibility criteria were determined by relevant elements of the PICOS guidance for undertaking literature reviews [23]. Papers were included if the population consisted of individuals with diabetes (type 1 or type 2), interventions and comparators was not applicable, outcomes consisted of the direct and indirect costs of diabetes and the study designs were observational or intervention studies, hypothetical studies and surveys. Articles were included if they: (1) were published between 2006 and 2016; (2) were in English language; (3) were published in peer-reviewed journals; (4) reported original research findings on diabetes COI or health expenditure data; (5) covered at least one African country as defined by the United Nations. Articles were excluded if they: (1) were economic evaluation studies that reported on costs derived from another study or publication; (2) only reported costs related to diabetes prevention; (3) were studies that did not provide original research or details on how costs were calculated; (4) did not provide patient specific costs; (5) were conference abstracts or poster presentations; (6) were animal studies.

### Extraction of information

In order to extract information from studies, we developed two extraction tables. One table was used to extract details such as year published, research objectives, study design and types of costs estimated. To present information on cost data across studies, we grouped the costs as follows: outpatient costs, inpatient costs, cost of drugs and combined costs. The combined costs category included studies in which inpatient and outpatient costs per patient were not separated. This category also included indirect costs due to loss of income, disability and premature mortality.

Two adjustments were made in the presentations of costs. First, in cases where relevant unit costs were not provided, these were calculated wherever possible using the information provided in the study. Second, in order to increase comparability of costs across studies we converted costs into international dollars (further denoted with the I$ sign) using the 2015 purchasing power parity (PPP) estimates. We followed the approach used by Seuring et al. [17]. Adjustments were made for each study in which costs were not presented in the country’s local currency. Costs for studies in which the United States Dollar (USD) exchange rate at the time of costing was provided, were adjusted using the provided exchange rate. For studies in which the exchange rate was not provided, we used the average exchange rate for the costing period based on the FX currency converter [24]. Costs presented in local currencies were immediately converted into international dollars. To estimate the PPP-adjusted costs, we then applied a cost converter web based tool developed by the Campbell and Cochrane Economics Methods Group (CCEMG) and the Evidence for Policy and Practice Information and Coordination Centre (EPPI Centre). Costs in their original costing year and local currency were first adjusted for inflation to give costs in 2015 and then converted to I$. Our study makes use of the IMF PPP method to make these adjustments to costs. For studies in which the costing year or year of data collection was not clear, we used the manuscript publication year as proxy. When a study was conducted over two different years (for example August 2012 to June 2013) we assumed the year of costing was the final year in which the study was concluded (i.e 2013). In multi-country analysis studies that do not provide individual country costs estimates and reported costs in USD we applied the GDP deflator to adjust for inflation.

The second table was used to list the technical criteria applied in assessing the quality of each study. A number of check lists have been developed for the evaluation of health economics studies, most of which focus on full economic evaluations such as cost benefit analysis, cost-effectiveness analysis and cost utility analysis [25]. Although various checklists have been developed for the appraisal of COI studies [14, 26–28] none of these checklists has been formally validated. The checklist used in this study is the one based on the ten point checklist for economic evaluation developed by Drummond [29] and later adapted to COI studies by Molinier et al. [14] and other COI studies [30–32]. We checked the quality of our systematic review based on the PRISMA 2009 checklist (see Additional file 2).

## Results

**Fig. 1:**
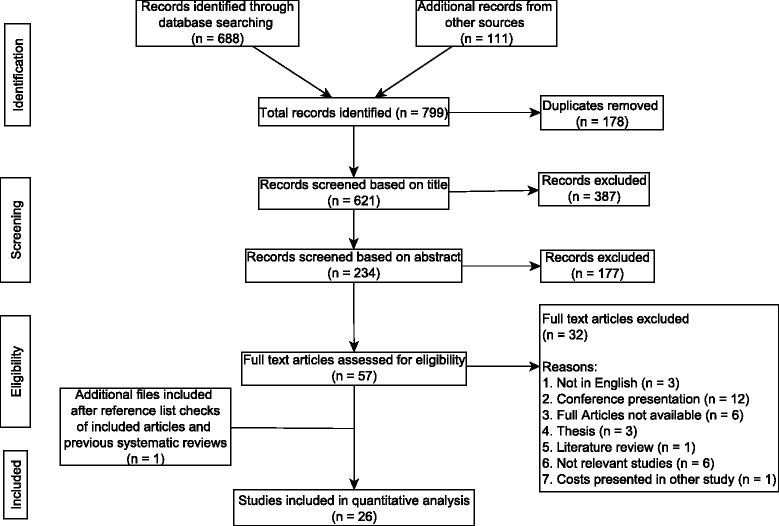
Flow chart of study selection process

The initial search strategy yielded a total of 799 articles, from which 178 duplicates were excluded. Of the remaining 621 articles, 387 were removed during the first level of title screening and 177 removed during the second level of abstract screening leaving 57 articles for full text evaluation. Of the 57 studies that underwent full text review, 32 were excluded for one of the following reasons: studies were not in English, were conference presentations, full text of articles was not available, were not peer reviewed, was a literature review of diabetes (not costs) in Nigeria, did not provide patient specific costs or presented costs that had been calculated in another included study (see Fig. 1). Twenty five studies were identified that met the inclusion criteria and one additional article that met the inclusion criteria, was identified after the reference screening giving the total of twenty-six articles included in the review.

### General characteristics of the included studies

The general characteristics of these twenty six studies are provided in Table 1. The list of articles is presented in Additional file 3 in alphabetical order of the author’s family name. A majority of the studies were published after the year 2010 (69%) and the rest between 2006 and 2010 (31%). Of the included studies, the year of costing was 2000–2005 for 7 studies (27%), between 2006 and 2010 for 6 studies (23%) between 2010 and 2016 for 9 studies (35%) and unclear for 4 studies (15%). It was also noticeable that most studies estimated the costs associated with diabetes for single countries (85%) and some included multiple countries (15%). Diabetes costs were mostly estimated for West African countries (*n* = 13) followed by East Africa (*n* = 6), Southern Africa (n-5), Northern Africa (*n* = 2) and Central Africa (n = 2). One study estimated costs for three groups of countries within the WHO African Region.

**Table 1:** General characteristics of studies included under review

Study characteristic	Number	Reference index in Additional file 3*
Year of publication		
2006–2010	8	5,8,9,11,13,18,22,25
After 2010	18	1,2,3,6,7,4,10,12,14,15,16,17,18,20,21,23,24,26
Year of costing		
2000–2005	7	8,9,11,13,18,21,24
2006–2010	6	1,6,12,14,17,22
After 2010	9	3,7,10,15,16,19,23,25,26
Not clear	4	2,4,5,20
No of African countries included in study		
One	22	1,4,5,6,7,8,9,10,11,12,14,15,16,17,18,19,21,22,23,25,25,26
More than one	4	2,3,13,19
Region		
Central Africa	2	14.19
Eastern Africa	6	5,6,11,16,19,23
Northern Africa	2	4, 8
Southern Africa	5	3,15,17,21,26
Western Africa	13	1,2,3,7,9,10,12,18,19,20,22,24,25
WHO African region	1	13
Cost Indicators		
Direct	26	1,2,3,4,5,6,7,8,9,10,11,12,13,14,15,16,17,18,19,20,21,22,23,24,25,26
Indirect costs	2	4.13
Perspective		
Not specified	10	4,5,7,8,9,11,16,17,21, 24
Family/patient	7	2,10,12,14,18,19,20
Societal	4	3,13,15,25
Health system/institution	2	22.23
Health system and patient	2	1.6
Government	1	26
DM type		
Type 1	1	19
Type 2	11	1,2,3,7,8,12,14,15,16,20,26
Type 1 and Type 2	9	4,5,10,11,13,18,22,24,25
Not specified	5	6,9,17,21,23
Sample sizes		
n.a	2	2.6
not specified	2	19.23
1–100	6	7,12,15,18,21,24
101–1000	12	1,8,9,10,11,14,16,17,20,22,25,26
1001–2000	1	5
> 1000 000	3	3,4,13
Epidemiological approaches		
Prevalence	25	1,2,4,5,6,7,8,9,10,11,12,13,14,15,16,17,18,19,20,21,22,23,24,25,26
Incidence	1	3
Study focus		
General costs	21	1,2,3,4,5,8,10,11,12,13,15,16,17,19,20,21,22,23,24,25,26
Diabetic foot ulcer	3	6,7,18
Drugs	2	9.14
Cost data source		
Hospital or medical centre	13	1,2,5,6,7, 9,11,17,19,21,22,23,24
Patients	3	8,16, 20
Not clear	3	14,18,25
Hospital plus other government institutions	1	12
Patients plus hospital	1	10
Who publications and various individual country services	1	13
Various data sources	4	3, 4, 15, 26

*One study can fall into more than one category

In conducting COI studies, previous literature reviews have suggested that it is important for the study to firstly define the illness, epidemiological sources, type of costs and study perspective. After which resource consumption and unit cost data can be collected and results presented along with sensitivity analysis [14, 30, 31]. These key methodological points are presented next.

### Defining the disease and population

Overall, the majority of the studies included in this review focused on the cost of type 2 diabetes (*n* = 11), nine studies considered the costs of both type 1 and type 2 diabetes, only one study considered the costs of type 1 diabetes, whilst five studies did not clearly define the type of diabetes assessed (Table 1). Eighty percent of the studies focused on the general costs of diabetes, 12 % focused on the diabetic complication of diabetic foot ulcer and 8 % focused on the cost of medication for diabetic individuals. In addition to discussing the general costs of diabetes, three studies also estimated the costs of diabetic complications including but not limited to hypertension, stoke and nephropathy [33–35]. The majority of the sample sizes ranged from 101 to 1000 (12 studies) followed by a sample size range of 1–100 (6 studies), > 1 million (3 studies) and 1001–2000 (1 study). Two studies did not provide the sample sizes [36, 37] and two studies were hypothetical and calculated costs without making use of any samples [33, 38]. In selecting the sample to be included in the analysis, eight studies specified the age [36, 39–43] and three studies indicated the duration of illness [40–42].

### Epidemiological approaches

Common epidemiological approaches in COI studies are the prevalence based approach and the incidence based approach [13]. The prevalence based approach is used to estimate the economic burden attributable to prevalent cases over a specific period usually one year. The incidence based approach involves analysis of the costs of diabetes within a given period [44]. The incidence based approach usually follows people at similar stages of disease or diagnosis. Whilst the study by Basu et al. [39] simulated the life course of present and new cases of diabetes over the period 2016–2025, the rest of the studies included in this review estimated the actual impact of existing cases over a period of 1 month to 2 years.

### Study perspective

COI studies can be done from various perspectives. Common perspectives are the patient perspective (for example OOP payments), the employer perspective (loss of productivity), health system perspective (hospital and primary care services), government perspective (infrastructure, support program costs) and societal perspective (loss of income while caring for the sick) [26]. The societal perspective is commonly applied due to its comprehensive nature. In this review, the most commonly adopted perspective was the patient perspective (7 studies) and respectively the societal perspective (*n* = 4), followed by the health system perspective (*n* = 2), the combined health system and patient perspective (*n* = 2) and the government perspective (*n* = 1). Ten studies did not mention what sort of perspective was adopted, however based on reviewer interpretation we were able to classify these (see Additional file 4: Table S1).

### Data resources

The majority of the studies used medical centers or hospital data as their cost data sources (13 studies). In three studies cost data were obtained from patient interviews [40, 42, 43], whilst one study used a combination of both patient and hospital cost data sources [41] and four studies made use of various data sources such as published field surveys, international drug price indicators, NGOs, health insurance data, healthcare service providers and supplier catalogs [39, 45, 54, 58]. One study that calculated the financial cost to families of children with type 1 diabetes, used cost data collected from IDF Life for a Child program centers [36].

### Resource quantification

The estimation of resource consumption can be prospectively or retrospectively performed [13]. In prospective COI studies, the event would not have occurred prior to the initiation of the study. Therefore prospective studies involve following up the patient over time. On the other hand, in retrospective studies, the events would have already occurred when the study is initiated. Three studies estimated costs prospectively [47–49], one study used a modeling approach [39], two studies were hypothetical [33, 38] and the rest of the studies estimated costs retrospectively. Eleven studies used a bottom-up approach in which either hospital records were reviewed [34, 35, 37, 49–56] or patient interviews were conducted [40–43, 48, 57] to gather activity data. In the study by Ogle et al. [36], resource use was based on the IDF Life for a Child program clinical experience. Expert opinion or standard practice was used to estimate resource consumption in some studies [33, 38, 39, 47, 58]. Resource consumption was estimated using various national indicators, national survey and published studies by Kirigia et al. [46] and Boutayeb et al. [45].

### Cost of diabetes mellitus

All studies included in the review provided sufficient information to calculate per capita costs and four studies extended the national costs of diabetes [35, 45, 50, 56]. The national direct costs of diabetes in Nigeria were estimated in the range of I$3.5 to I$4.5 billion per annum [35, 50, 56] whilst in Morocco the estimated national costs (direct and indirect) are in the range I$5.9 to I$8.2 billion per annum [45]. Two studies under review quantified both direct and indirect costs [45, 46]. The indirect costs were those costs associated with the loss of income, disability and premature mortality. In both studies, the human capital approach was applied in measuring costs. In both studies, the general consensus was that permanent disability accounted for the largest portion of the indirect costs. The studies that provided both direct and indirect costs show that indirect costs were higher than direct costs (Additional file 4: Table S1).

The direct costs considered in the studies under review were medical and non-medical costs. Detailed costing of the studies under review is provided in Additional file 4: Table S1. The table groups costs according to outpatient, inpatient and combined costs (i.e. outpatient and inpatient). Twelve studies presented outpatient costs per individual per annum [33, 35, 36, 39, 40, 43, 47, 50, 55–58]. It is important to note that during the estimation, cost studies used various healthcare components in calculating costs; therefore results are not directly comparable. In calculating direct costs, the most commonly included healthcare items were drug costs, followed by diagnostic costs, medical supply or disposable costs and consultation costs, just to name a few (see Additional file 4: Table S1). The presentation of out-patient costs varied across the studies. In order to facilitate comparability we converted costs to per capita costs. Therefore, unless indicated otherwise, outpatient costs presented in Additional file 4: Table S1 are average costs per individual per annum. From Additional file 4: Table S1 no linear increase in costs is visible between 2002 and 2016 for individual country estimates. For example the direct per capita out-patient costs in Nigeria varied from I$1143 in 2004 to I$616 in 2012. Costs also varied widely between countries. Wide differences are also observed for costs across the various country income groups in the study. The wide variation in costs is to a great extent a result of differences in costing methods and cost categories included in the cost estimation. When assessing costs in which costing methods applied and costing components included were similar, it is observed that Burkina Faso generally had higher out-patients costs, followed by Mali, Benin and then Guinea [33]. Hospitalization costs also varied significantly within and across countries. For South Africa a notable increase in costs is visible between 2005 and 2009 (I$ 1813 to I$6871). Once again, the costs presented in Additional file 4: Table S1 are however not directly comparable due to differences in costing methods and cost components included in the calculation of costs.

One crucial challenge highlighted in some of the studies was the OOP expenses incurred by patients. This challenge has also been reported elsewhere [8, 59]. OOP healthcare costs are a hindrance to healthcare access and could lead to catastrophic health expenditure and impoverishment [4]. According to the World Health Organization African region 2014 expenditure atlas, catastrophic health expenditure is low in countries where OOP expenditure is below 20% of total health expenditure [59]. Where data is available, Additional file 4: Table S1 also presents data from the World Bank showing OOP expenditure as a percentage of total health expenditure in each country. As shown in Additional file 4: Table S1, in 2014 South Africa was the only country in which OOP expenditure was less than 20%. This is very concerning given that diabetes is a chronic illness that requires frequent healthcare access [2].

The cost of drugs was reported separately in a majority of the studies (65%). In studies reporting both drug costs and total costs of treatment, drug costs often took up a significant portion of total costs of treatment costs, 14% - 90% in Nigeria, 64% in Ethiopia, 53% in Sudan, 14% in the Seychelles, 4% to 7% in South Africa and 5% in Uganda (see Table 2). Other cost components that were commonly reported separately were diagnostic costs (*n* = 12), transportation costs (*n* = 9), and consultation costs (*n* = 7). Less than four studies reported on the costs associated with each of the following healthcare components separately; surgery, insurance premiums, service costs, physiotherapy, disposables, personnel, capital and diabetic diet costs.

**Table 2:** Drug costs in diabetic patients

Ref*	Country	DM Type	Drug costs (I$)	% of treatment cost
Outpatient				
1	Nigeria	2	468.28 /annum p.p	62%
5	Seychelles	1 & 2	6.09 / annum p.p	14%
8	Sudan	2	42,177.93 /annum p.p	53%
10	Nigeria	1 & 2	80.97 / month p.p	77%
12	Nigeria	2	362.41 / patient / annum	39%
20	Nigeria	2	88 / month p.p	14%
23	Uganda	n.s	0.36 / visit	5%
24	Nigeria	1 & 2	1025 / annum p.p	90%
25	Nigeria	1 & 2	420.63 / annum p.p	68%
Hospitalisation				
7	Nigeria	2	553.03 / admission	47%
11	Ethiopia	1 & 2	511.83 / person / admission	64%
17	South Africa	n.s	461 / patient / admission	7%
18	Nigeria	1 & 2	1438 / admission	50%
21	South Africa	n.s	80 / person / admission	4%
Others				
9	Nigeria	n.s	4.81 / patient /day	n.a
14	Cameroon	2	3.85 / person / month	n.a
16	Kenya	2	Insulin - 10 / month / p.p (average cost) Oral agents - 20 / month / p.p (median cost)	n.a

Notes: *DM* diabetes mellitus, *p.p* per person, *n.a* not applicable, *n.s* not specified

*Reference index in Additional file 3

Three studies that separately provided the costs associated with type 1 diabetes mellitus (T1DM) and type 2 diabetes mellitus (T2DM) show that the direct costs of T1DM were higher than T2DM [35, 55, 56]. The hospital based studies showed that the excess cost ratio between T1DM and T2DM ranged from 1.80 to 5.66.The excess cost of diabetics versus non-diabetics are reported in two studies [34, 48]. Both studies calculated hospitalization costs and found the hospitalization costs for diabetic patients to be higher than non-diabetic patients; a cost ratio of 1.27 to 1.50.

Five studies included in the review estimated the costs of specific types of complications [33, 35, 38, 51, 60]. Three of these studies focused primarily on estimating the costs associated with the diabetes complication of foot ulcer [38, 51, 60]. The costs associated with the treatment of diabetic foot ulcer in the studies varied significantly, depending on the stage of severity of the illness (Additional file 4: Table S1). The excess costs of diabetes complications versus no complications were reported in two studies [33, 35]. Both studies showed that complications increased the burden of diabetes significantly. The incremental costs were reported for various types of complications and various countries. Among the complications investigated, the highest average cost ratios were recorded for nephropathy, diabetic foot and acute stroke. The lowest cost ratios were recorded for retinopathy, keto acidosis and hypertension. These costs details are provided in an additional file (see Additional file 5).

A few other studies investigated the cost of diabetes in relation to income levels, age and by comparing costs of public versus private healthcare providers. Studies found that the highest burden of diabetes was amongst individuals of low socio-economic status [43, 57]. Okoronkwo et al. used an asset based socio-economic index to group the sample into four quartiles. The authors found that the economic burden of T2DM was highest in individuals that fell within the lowest socio-economic quartile [43]. Ipingbemi and Erhun found that the economic burden of diabetes was highest in respondents who earned less that USD 125 per month [57]. Whilst investigating the costs associated with diabetes in relation to age, three studies found different results. A Nigerian study by Ipingbemi and Erhun found the average outpatient cost of diabetes was highest amongst those within the age group 60 to 69 years [57]. A study in Sudan finds that the outpatient cost is highest in those above 60 years of age [40]. Mutowo et al. find that hospitalization costs were lower in those older than 65 [54]. Alouki et al. and Elrayah-Eliadarous et al. both found that medical care in the public sector was less costly when compared to the private sector [33, 40].

### Quality of the included studies

Most studies presented and explained their results in a clear way, consistent with the methodology of the study. Presentation of results was generally in agreement with the study aim and the conclusions were made in line with the results presented. More than 70% of the included studies carefully described the epidemiological sources, activity data, and unit costs. A few studies (24%) included in this review did not discuss any limitations regarding the methodologies employed in calculating costs. A common weakness discussed was that studies that collected data from patient interviews were subject to recall and social desirability bias. Also, the use of one study site and small sample sizes meant that results were not applicable to other sites or national estimates. The studies by Boutayeb et al. [45] and Kirigia et al. [46] were also limited by factors such as the assumptions concerning the number of individuals using insulin or oral drugs, the number of people using outpatient or inpatient services, the number of diagnostic tests conducted. Hypothetical studies included in the review were subject to unavoidable differences in the treatment options provided by working groups, expert advice or physicians [33, 38, 39].

Costs were discounted in three studies and the discount rate chosen was 3%. However, the discount rate chosen was not explained [37, 39, 46]. In the rest of the studies, the time horizon was short (<2 years) and therefore, costs were not discounted. Sensitivity analysis was conducted in three studies. A one-way simple sensitivity analysis was conducted in one study [55], another study conducted six sensitivity analysis [39] and the last one used probabilistic tests [58].

Based on the methodological points discussed above, Table 3 shows the proportion of studies that met the criteria for the reporting of COI used in this review. For most of the studies (60%) the answer was yes in 6 to 10 questions asked. It should be noted that the checklist has been adapted to the needs of this review and questions were benchmarked against the objectives of the study under review. A few studies did not clearly articulate or provide explicit information regarding the methodologies followed [38, 60]. In some studies, the primary objective of the study was not to estimate costs hence it did not clearly explain methods adopted for cost estimation [47, 53, 58].

**Table 3:** Quality index score for studies included in review

Question	Reference index in Additional file 3																									
	1	2	3	4	5	6	7	8	9	10	11	12	13	14	15	16	17	18	19	20	21	22	23	24	25	26
1. Was a clear definition of the illness given?	+	√	√	+	√	+	√	+	+	√	√	√	+	√	√	√	+	√	√	+	+	+	x	√	√	+
2. Were epidemiological sources carefully described?	+	n.a	√	√	+	n.a	√	√	√	√	√	√	√	√	√	√	√	√	+	√	√	√	x	√	√	x
3. Were costs sufficiently disaggregated?	√	√	√	+	+	+	+	+	x	√	+	√	√	√	√	+	+	√	√	+	+	√	√	+	√	+
4. Were activity data appropriately assessed?	√	√	+	√	√	+	√	√	√	√	√	√	√	+	√	√	√	+	√	√	√	√	√	√	√	√
5. Were the sources of all cost values analytically described	√	+	√	+	+	+	+	√	√	+	√	√	√	+	+	√	√	+	√	√	√	√	√	√	√	+
6. Were unit costs appropriately valued?	+	√	√	√	√	√	√	+	√	+	√	+	√	+	√	√	√	+	√	√	√	+	√	√	√	√
7. Were the methods adopted carefully explained?	√	√	√	√	+	+	+	√	√	√	+	+	√	+	+	√	√	+	+	+	√	√	√	√	√	+
8. Were costs discounted	n.a	n.s	+	n.a	n.a	n.a	n.a	n.a	n.a	n.a	n.a	n.a	+	n.a	n.a	n.a	n.a	n.a	n.a	n.a	n.a	n.a	+	n.a	n.a	n.a
9. Were the major assumptions tested in a sensitivity analysis?	x	x	√	x	x	x	x	x	x	x	x	x	x	x	x	x	x	x	x	x	x	√	x	x	x	√
10. Was the presentation of study results consistent with the methodology of the study?	√	√	√	√	√	+	√	√	+	√	√	√	√	√	√	√	√	√	√	√	√	√	√	√	√	√
Total score by study																										
√ Yes	5	6	8	5	4	1	5	5	5	6	6	6	7	4	6	7	6	4	6	5	6	7	6	7	8	4
+ partially	3	1	2	3	4	6	3	3	2	2	2	2	2	4	2	1	2	4	2	3	2	2	1	1	0	4
x No	1	1	0	1	1	1	1	1	2	1	1	1	1	1	1	1	1	1	1	1	1	0	3	1	1	1
n.a not applicable	1	1	0	1	1	2	1	1	1	1	1	1	0	1	1	1	1	1	1	1	1	1	0	1	1	1

## Discussion

There has been growing concern over the increase in the prevalence and burden of NCDs. Endorsements such as the Moscow Declaration on NCDs in May 2011 and the Political Declaration on the prevalence and control of NCDs in September 2011 have been instrumental in raising awareness on the urgent need to place NCDs on the government agendas [61, 62]. Following this some countries in Africa such as Ghana and South Africa have drawn up national policies and plans for the prevention and control of NCDs [63, 64]. More recently the WHO Global conference on NCDs in Uruguay decisively raised priority on the need for a multi-sectoral approach and policy coherence in order to meet the targets set out by SDG 3. All these developments are testament to the awareness surrounding the burden of NCDs such as diabetes. African countries are faced with challenges in addressing the rise in NCDs whilst still grappling with infectious diseases [2]. This threatens to overwhelm an already overstretched healthcare sector and pose a challenge to economic development in Africa [8]. There is thus growing need to assess the burden associated with these NCDs in Africa and prioritize interventions that prevent or delay the onset of diabetes.

This study contributes to the understanding of the costs of diabetes in Africa and the methods used to estimate these costs. The first objective of this review was to identify and capture the evidence presented in the literature on the overall direct and indirect costs of diabetes in Africa that have been published since 2006. The literature search identified 26 studies that met the eligibility criteria of this review. Our findings suggested that the annual economic burden of diabetes in Africa was huge. Most of these healthcare costs in Africa are borne by the patients and this influences the attainment of proper care due to financial constraints [8]. These costs are compounded by the presence of complications which often arise as a result of the presence of risk factors [51]. Two studies included in our review that calculate indirect costs of diabetes show that the indirect costs are higher than the direct costs. This finding is contrary to the results from a global COI study on diabetes by Seuring et al. which finds that the direct costs associated with diabetes are higher than the indirect costs [17]. It should however be noted that whilst our finding is based on only two studies that estimated indirect costs the finding by Seuring et al. is based on evidence from a much larger group of publications (*n* = 26). Seuring et al. find that direct costs are much higher than indirect costs mostly in high income countries [17].

Similar to a systematic review conducted by Seuring et al. [17], we also find that many studies did not specify the type of diabetes being reviewed. This makes it difficult to isolate and document the burden of T2DM which is the most common type of diabetes in Africa [6] and is largely avoidable [10]. Not specifying the type of diabetes also makes it difficult to make comparisons in the differences in costs due to the different types of diabetes. However based on the three studies that separated the costs associated with T1DM and T2DM the cost of T1DM is higher than T2DM. Whilst our study finds a cost ratio of between 1.80 to 5.66 Ng et al. find a cost ratio of 1.5 to 4.4 in their systematic review [16]. The cost ratios for diabetes complications versus no complications ranged from 1.9 to 2.1 in Ng et al. [16] whilst our review found a cost ratio of between 1.08 to 4.38. Whilst Ng et al. included a much larger group of studies; our review only includes a few articles that report on the cost of T1DM versus T2DM and diabetes complications versus no complications [16].

Like other diabetes COI reviews drug costs, diagnostic costs and consultation costs are some of the most commonly reported items [16, 17, 65]. Most studies that reported total treatment costs and cost of drugs showed that the cost of drugs weighed heavily upon the burden of diabetes, accounting for a significant portion of all direct costs [35, 40, 41, 48, 50, 56, 60]. This finding is similar to Yesudian et al. [65] who found that drug cost often constitutes 50% of the total direct costs. Many studies included in our review note that the reason for such high costs of drugs is that physicians commonly prescribe branded products. The integration of generic medicines in the writing of prescriptions is therefore crucial to reduce diabetes costs in Africa [41, 50, 52].

Amongst the studies that reported on the cost of diabetes across various socioeconomic parameters such as income, the general consensus was that the economic burden of diabetes weighed more heavily upon those in the low income group [57] or low socio-economic status [43]. This finding is consistent with a literature review on the economic impact of diabetes in India, which also find that lower income groups generally spent more on diabetes healthcare [65].

Our second objective was to review the methods that have been used to calculate the costs of diabetes in Africa and assess the quality of the COI studies that have been included in the review. A majority of the studies were prevalence based which is considered to be the most suitable for measuring costs related to chronic diseases [26]. This finding is also consistent with previous COI studies in diabetes by Yesudian et al. and Ng et al. who find that a majority of the COI studies included in their review, employed a prevalence based study design [16, 65]. A majority of the studies were hospital based studies involving small sample sizes; as such results might not be applicable to national estimates. A major limitation of most studies is that sensitivity analysis was not performed. The studies analyzed, applied various methodologies in the estimation of cost, hence it is very difficult to generalize results or compare the results from different studies. The differences in the methodologies applied are probably due to an absence of a formal validated methodology to be followed in COI studies [14]. According to the key methodological questions asked for most of the studies, the answer was yes in 6 to 10 questions asked (64%) indicating an adequate quality assessment for most of the studies.

From this review it is clear that national estimates on the economic impact of diabetes in African countries are lacking. Therefore this review provides a fragmented picture of the economic impact of diabetes in Africa. In addition, a few studies estimated the indirect costs of diabetes. Although diabetes potentially affects the ability of individuals to participate in the labor market, evidence on labor markets effects is lacking. The estimates provided on the cost of diabetes did not take into account undiagnosed diabetes. As a result, it is highly likely that the aggregate costs associated with diabetes have been severely under estimated. Studies that provide national estimates of unmet diabetes are important for carefully estimating the national impact of the disease.

### Limitations

The current review has some limitations. The exclusion of articles not written in English could have led to the omission of relevant articles in the area under study. The review also focused on peer reviewed articles and excluded grey literature such as academic thesis. The exclusion of this literature might introduce some bias into the review. A broader search without this restriction would yield different results. Like many other checklists, the results presented in the checklist may not be directly replicable since the results are a subjective assessment by the reviewer. In addition the checklist used in this review does not give weighting scores on the various items included in the list. As a result all items are given equal scoring although some items influence results more than others [30]. Due to the heterogeneity in study designs and methods used in the estimation of costs, the costs presented in the study are not comparable. As a result it was not possible to conduct a meta-analysis.

## Conclusion

There has been a growing global focus on NCDs since the United Nations High Level Meeting on NCDSs in 2011. More recently, the specific inclusion of NCDs in the SDGs provides African countries with an opportunity to directly focus on and increase the momentum in tackling NCDs. SDG3 makes a commitment to reduce pre mature mortality in NCDs, to eliminate inequalities in healthcare costs and protect patients from diabetes induced catastrophic health expenditure, amongst others. In order to accelerate the progress in meeting SDG3 set for 2030 consideration must also be given to promoting early detection and monitoring the prevalence of NCD risk factors. The adoption of policies targeted at a reduction in harmful alcohol use, tobacco consumption and physical inactivity will contribute to a reduction in NCD prevalence and therefore healthcare costs.

This review provides a snapshot of the costs and economic burden of diabetes in Africa. We found that many studies on the cost of diabetes were conducted in West Africa and focused on the direct costs. This provides the West African region sufficient information to act on establishing cost effective treatments that could influence a reduction in cost. Few studies estimated the economic impact of the disease using the societal perspective and few studies estimated the costs of diabetes at the national level. A common conclusion in the studies included under the review is that diabetes imposes a considerable economic burden on individuals and that amongst the cost components included in cost calculation, the cost of drugs imposed the largest burden on total costs incurred. These areas call for policies that focus on a reduction in the burden of diabetes on individuals and the promotion of prescriptions of diabetes drugs in their generic names. As the prevalence of diabetes in Africa is expected to rise, the economic burden on individuals will also continue to increase. COI studies are vital in providing information that supports economic evaluations and policy development. Future research should focus on increasing the transparency and methodological principles of COI studies.

## Additional files

Additional file 1: Search strategy. (DOCX 14 kb)

Additional file 2: PRISMA Checklist. (DOCX 18 kb)

Additional file 3: Articles included in review in alphabetical order. (DOC 27 kb)

Additional file 4: Table S1. Components of direct and indirect costs for diabetes mellitus and reflecting cost. (DOCX 52 kb)

Additional file 5: Cost ratio for individuals with and without diabetes mellitus complications (DOCX 21 kb)

## Abbreviations

COI: Cost of Illness
GDP: Gross Domestic Product
IDF: International Diabetes Federation
NCD: Non-Communicable Diseases
OOP: Out of pocket
PPP: Purchasing Power Parity
PRISMA: Preferred Reporting Items for Systematic Reviews and Meta-Analysis
T1DM: Type 1 Diabetes Mellitus
T2DM: Type 2 Diabetes Mellitus
USD: United States Dollar
WHO: World Health Organisation
