# Supplementary material for: Cost of diabetes mellitus in Africa: a systematic review of existing literature
Source: Global Health. 2018 Jan 16;14:3. doi: 10.1186/s12992-017-0318-5 (PMC5771003; doi:10.1186/s12992-017-0318-5)
Supplement: Supplementary file 1 — Search strategy. (DOCX 14 kb) [file 12992_2017_318_MOESM1_ESM.docx]

**Additional file 1 – Search strategy**

**PubMed**

((("diabetes mellitus"[MeSH Terms] OR ("diabetes"[All Fields] AND "mellitus"[All Fields]) OR "diabetes mellitus"[All Fields] OR "diabetes"[All Fields] OR "diabetes insipidus"[MeSH Terms] OR ("diabetes"[All Fields] AND "insipidus"[All Fields]) OR "diabetes insipidus"[All Fields]) OR "diabetes mellitus"[All Fields] OR diabetics[All Fields]) AND (("economics"[Subheading] OR "economics"[All Fields] OR "cost"[All Fields] OR "costs and cost analysis"[MeSH Terms] OR ("costs"[All Fields] AND "cost"[All Fields] AND "analysis"[All Fields]) OR "costs and cost analysis"[All Fields]) OR "cost of illness"[All Fields] OR "economic burden"[All Fields] OR ("health expenditures"[MeSH Terms] OR ("health"[All Fields] AND "expenditures"[All Fields]) OR "health expenditures"[All Fields] OR "expenditure"[All Fields]) OR "healthcare costs"[All Fields])) AND (("africa"[MeSH Terms] OR "africa"[All Fields]) OR ("africa south of the sahara"[MeSH Terms] OR ("africa"[All Fields] AND "south"[All Fields] AND "sahara"[All Fields]) OR "africa south of the sahara"[All Fields] OR ("sub"[All Fields] AND "saharan"[All Fields] AND "africa"[All Fields]) OR "sub saharan africa"[All Fields]) OR ("angola"[MeSH Terms] OR "angola"[All Fields]) OR ("benin"[MeSH Terms] OR "benin"[All Fields]) OR ("botswana"[MeSH Terms] OR "botswana"[All Fields]) OR ("burkina faso"[MeSH Terms] OR ("burkina"[All Fields] AND "faso"[All Fields]) OR "burkina faso"[All Fields]) OR ("burundi"[MeSH Terms] OR "burundi"[All Fields]) OR ("cape verde"[MeSH Terms] OR ("cape"[All Fields] AND "verde"[All Fields]) OR "cape verde"[All Fields] OR ("cabo"[All Fields] AND "verde"[All Fields]) OR "cabo verde"[All Fields]) OR ("cameroon"[MeSH Terms] OR "cameroon"[All Fields]) OR ("central african republic"[MeSH Terms] OR ("central"[All Fields] AND "african"[All Fields] AND "republic"[All Fields]) OR "central african republic"[All Fields]) OR ("chad"[MeSH Terms] OR "chad"[All Fields]) OR ("comoros"[MeSH Terms] OR "comoros"[All Fields]) OR ("congo"[MeSH Terms] OR "congo"[All Fields]) OR ("cote d'ivoire"[MeSH Terms] OR ("cote"[All Fields] AND "d'ivoire"[All Fields]) OR "cote d'ivoire"[All Fields]) OR ("democratic republic of the congo"[MeSH Terms] OR ("democratic"[All Fields] AND "republic"[All Fields] AND "congo"[All Fields]) OR "democratic republic of the congo"[All Fields]) OR ("djibouti"[MeSH Terms] OR "djibouti"[All Fields]) OR ("egypt"[MeSH Terms] OR "egypt"[All Fields]) OR ("equatorial guinea"[MeSH Terms] OR ("equatorial"[All Fields] AND "guinea"[All Fields]) OR "equatorial guinea"[All Fields]) OR ("eritrea"[MeSH Terms] OR "eritrea"[All Fields]) OR ("ethiopia"[MeSH Terms] OR "ethiopia"[All Fields]) OR ("gabon"[MeSH Terms] OR "gabon"[All Fields]) OR ("gambia"[MeSH Terms] OR "gambia"[All Fields]) OR ("ghana"[MeSH Terms] OR "ghana"[All Fields]) OR ("guinea"[MeSH Terms] OR "guinea"[All Fields]) OR ("guinea-bissau"[MeSH Terms] OR "guinea-bissau"[All Fields] OR ("guinea"[All Fields] AND "bissau"[All Fields]) OR "guinea bissau"[All Fields]) OR ("kenya"[MeSH Terms] OR "kenya"[All Fields]) OR ("lesotho"[MeSH Terms] OR "lesotho"[All Fields]) OR ("liberia"[MeSH Terms] OR "liberia"[All Fields]) OR ("libya"[MeSH Terms] OR "libya"[All Fields]) OR ("madagascar"[MeSH Terms] OR "madagascar"[All Fields]) OR ("malawi"[MeSH Terms] OR "malawi"[All Fields]) OR ("mali"[MeSH Terms] OR "mali"[All Fields]) OR ("mauritania"[MeSH Terms] OR "mauritania"[All Fields]) OR ("mauritius"[MeSH Terms] OR "mauritius"[All Fields]) OR ("comoros"[MeSH Terms] OR "comoros"[All Fields] OR "mayotte"[All Fields]) OR ("morocco"[MeSH Terms] OR "morocco"[All Fields]) OR ("mozambique"[MeSH Terms] OR "mozambique"[All Fields]) OR ("namibia"[MeSH Terms] OR "namibia"[All Fields]) OR ("niger"[MeSH Terms] OR "niger"[All Fields]) OR ("nigeria"[MeSH Terms] OR "nigeria"[All Fields]) OR ("reunion"[MeSH Terms] OR "reunion"[All Fields]) OR ("rwanda"[MeSH Terms] OR "rwanda"[All Fields]) OR ("atlantic islands"[MeSH Terms] OR ("atlantic"[All Fields] AND "islands"[All Fields]) OR "atlantic islands"[All Fields] OR ("saint"[All Fields] AND "helena"[All Fields]) OR "saint helena"[All Fields]) OR ("atlantic islands"[MeSH Terms] OR ("atlantic"[All Fields] AND "islands"[All Fields]) OR "atlantic islands"[All Fields] OR ("sao"[All Fields] AND "tome"[All Fields] AND "principe"[All Fields]) OR "sao tome and principe"[All Fields]) OR ("senegal"[MeSH Terms] OR "senegal"[All Fields]) OR ("seychelles"[MeSH Terms] OR "seychelles"[All Fields]) OR ("sierra leone"[MeSH Terms] OR ("sierra"[All Fields] AND "leone"[All Fields]) OR "sierra leone"[All Fields]) OR ("somalia"[MeSH Terms] OR "somalia"[All Fields]) OR ("south africa"[MeSH Terms] OR ("south"[All Fields] AND "africa"[All Fields]) OR "south africa"[All Fields]) OR ("south sudan"[MeSH Terms] OR ("south"[All Fields] AND "sudan"[All Fields]) OR "south sudan"[All Fields]) OR ("sudan"[MeSH Terms] OR "sudan"[All Fields]) OR ("swaziland"[MeSH Terms] OR "swaziland"[All Fields]) OR ("tanzania"[MeSH Terms] OR "tanzania"[All Fields]) OR ("togo"[MeSH Terms] OR "togo"[All Fields]) OR ("tunisia"[MeSH Terms] OR "tunisia"[All Fields]) OR ("uganda"[MeSH Terms] OR "uganda"[All Fields]) OR ("zambia"[MeSH Terms] OR "zambia"[All Fields]) OR ("zimbabwe"[MeSH Terms] OR "zimbabwe"[All Fields])) AND (("2006/01/01"[PDAT] : "2016/12/31"[PDAT]) AND English[lang])
