# Supplementary material for: Cost of diabetes mellitus in Africa: a systematic review of existing literature
Source: Global Health. 2018 Jan 16;14:3. doi: 10.1186/s12992-017-0318-5 (PMC5771003; doi:10.1186/s12992-017-0318-5)
Supplement: Supplementary file 3 — Articles included in review in alphabetical order. (DOC 27 kb) [file 12992_2017_318_MOESM3_ESM.doc]

**Additional file 3: Articles included in review in alphabetical order:**

1. Abdulganiyu G, Fola T. What is the cost of illness of type II Diabetes Mellitus in a Developing Economy. Int J Pharm Pharm Sci. 2014;6:927-31.

2. Alouki K, Delisle H, Besançon S, Baldé N, Sidibé-Traoré A, Drabo J, et al. Simple calculator to estimate the medical cost of diabetes in sub-Saharan Africa. World journal of diabetes. 2015;6(16):1312.

3. Basu S, Shankar V, Yudkin JS. Comparative effectiveness and cost-effectiveness of treat-to-target versus benefit-based tailored treatment of type 2 diabetes in low-income and middle-income countries: a modelling analysis. The Lancet Diabetes & Endocrinology. 2016;4(11):922-32.

4. Boutayeb W, Lamlili ME, Boutayeb A, Boutayeb S. Estimation of direct and indirect cost of diabetes in Morocco. 2013.

5. Bovet P, Shamlaye C, Gabriel A, Riesen W, Paccaud F. Prevalence of cardiovascular risk factors in a middle-income country and estimated cost of a treatment strategy. BMC Public health. 2006;6(1):9.

6. Cavanagh P, Attinger C, Abbas Z, Bal A, Rojas N, Xu ZR. Cost of treating diabetic foot ulcers in five different countries. Diabetes/metabolism research and reviews. 2012;28(S1):107-11.

7. Danmusa UM, Terhile I, Nasir IA, Ahmad AA, Muhammad HY. Prevalence and healthcare costs associated with the management of diabetic foot ulcer in patients attending Ahmadu Bello University Teaching Hospital, Nigeria. International journal of health sciences. 2016;10(2):219.

8. Elrayah-Eliadarous H, Yassin K, Eltom M, Abdelrahman S, Wahlström R, Östenson C-G. Direct costs for care and glycaemic control in patients with type 2 diabetes in Sudan. Experimental and clinical endocrinology & diabetes. 2010;118(04):220-5.

9. Enwere O, Salako B, Falade C. Prescription and cost consideration at a diabetic clinic in Ibadan, Nigeria: A report. Annals of Ibadan postgraduate medicine. 2006;4(2).

10. Fadare J, Olamoyegun M, Gbadegesin B. Medication adherence and direct treatment cost among diabetes patients attending a tertiary healthcare facility in Ogbomosho, Nigeria. Malawi Medical Journal. 2015;27(2):65-70.

11. Feleke Y, Enquselassie F. Cost of hospitalization of diabetic patients admitted at Tikur Anbessa Specialized Hospital, Addis Ababa, Ethiopia. Ethiopian medical journal. 2007;45(3):275-82.

12. Ipingbemi A, Erhun W. Cost implications of treatment of diabetes mellitus in a secondary healthcare facility in Ibadan. African journal of medicine and medical sciences. 2015;44(1):79-87.

13. Kirigia JM, Sambo HB, Sambo LG, Barry SP. Economic burden of diabetes mellitus in the WHO African region. BMC international health and human rights. 2009;9(1):1.

14. Labhardt ND, Balo JR, Ndam M, Manga E, Stoll B. Improved retention rates with low-cost interventions in hypertension and diabetes management in a rural African environment of nurse-led care: a cluster-randomised trial. Tropical Medicine & International Health. 2011;16(10):1276-84.

15. Mutowo MP, Lorgelly PK, Laxy M, Renzaho A, Mangwiro JC, Owen AJ. The Hospitalization Costs of Diabetes and Hypertension Complications in Zimbabwe: Estimations and Correlations. Journal of diabetes research. 2016;2016.

16. Mwavua SM, Ndungu EK, Mutai KK, Joshi MD. A comparative study of the quality of care and glycemic control among ambulatory type 2 diabetes mellitus clients, at a Tertiary Referral Hospital and a Regional Hospital in Central Kenya. BMC research notes. 2016;9(1):12.

17. Ncube-Zulu T, Danckwerts MP. Comparative hospitalization cost and length of stay between patients with and without diabetes in a large tertiary hospital in Johannesburg, South Africa. International Journal of Diabetes in Developing Countries. 2014;34(3):156-62.

18. Ogbera A, Fasanmade O, Ohwovoriole A, Adediran O. An assessment of the disease burden of foot ulcers in patients with diabetes mellitus attending a teaching hospital in Lagos, Nigeria. The international journal of lower extremity wounds. 2006;5(4):244-9.

19. Ogle G, Kim H, Middlehurst A, Silink M, Jenkins A. Financial costs for families of children with Type 1 diabetes in lower-income countries. Diabetic Medicine. 2015.

20. Okoronkwo IL, Ekpemiro JN, Okwor EU, Okpala PU, Adeyemo FO. Economic burden and catastrophic cost among people living with type2 diabetes mellitus attending a tertiary health institution in south-east zone, Nigeria. BMC research notes. 2015;8(1):527.

21. Pepper D, Levitt N, Cleary S, Burch V. Hyperglycaemic emergency admissions to a secondary-level hospital-an unnecessary financial burden: original article. South African Medical Journal. 2007;97(10):963-7.

22. Quaye EA, Amporful EO, Akweongo P, Aikins MK. Analysis of the financial cost of diabetes mellitus in four Cocoa clinics of Ghana. Value in Health Regional Issues. 2015;7:49-53.

23. Settumba SN, Sweeney S, Seeley J, Biraro S, Mutungi G, Munderi P, et al. The health system burden of chronic disease care: an estimation of provider costs of selected chronic diseases in Uganda. Tropical medicine & international health. 2015;20(6):781-90.

24. Suleiman I, Fadeke O, Okubanjo O. Pharmacoeconomic evaluation of anti-diabetic therapy in a Nigerian Tertiary Health Institution. Annals of African medicine. 2006;5(3):132-7.

25. Suleiman IA, Festus JA. Cost of illness among diabetes mellitus patients in Niger Delta, Nigeria. Journal of Pharmaceutical Health Services Research. 2015;6(1):53-60.

26. Volmink HC, Bertram MY, Jina R, Wade AN, Hofman KJ. Applying a private sector capitation model to the management of type 2 diabetes in the South African public sector: a cost-effectiveness analysis. BMC health services research. 2014;14(1):444.
