# Supplementary material for: Cost of diabetes mellitus in Africa: a systematic review of existing literature
Source: Global Health. 2018 Jan 16;14:3. doi: 10.1186/s12992-017-0318-5 (PMC5771003; doi:10.1186/s12992-017-0318-5)
Supplement: Supplementary file 4 — Components of direct and indirect costs for diabetes mellitus and reflecting cost. (DOCX 52 kb) [file 12992_2017_318_MOESM4_ESM.docx]

Additional file 4: Table S2 Components of direct and indirect costs for diabetes mellitus and reflecting cost

|  | |  | **COMMON COST COMPONENTS** ^i^ | | | | | | | | | | | | | | | | | | **COMORBI- DITIES** | | | |  |  | |  | |  |  | |  |  |
| --- | --- | --- | --- | --- | --- | --- | --- | --- | --- | --- | --- | --- | --- | --- | --- | --- | --- | --- | --- | --- | --- | --- | --- | --- | --- | --- | --- | --- | --- | --- | --- | --- | --- | --- |
|  |  |  | Not specified | Consultation | Admission | Theatre procedures | Transport | Drugs | Diagnostic | Insurance premium | Medical supplies/Disposables | Service costs | Personnel costs | Capital cost | Extra house keeper/caregiver | Physiotherapy | Food | Loss of income | Permanent disability | Premature mortality | Not specified | Yes, but not specified | Yes and specified | No |  |  | |  | |  |  | |  | |
| **Ref*** | **Country** | **Costing date** |  |  |  |  |  |  |  |  |  |  |  |  |  |  |  |  |  |  |  |  |  |  | **DM Type** | | **Perspective** | | **Direct (Indirect) Costs** ^a^ (I$) | | | **OOP (% of THE)** |  |  |
| **OUTPATIENT COSTS** | | | | | | | | | | | | | | | | | | | | | | | | | | | | | | | |  |  |  |
| (1) | Nigeria | 2010 |  |  |  |  | √ | √ | √ |  |  |  | √ |  |  |  |  |  |  |  |  | √ |  |  | 2 | | Health system & patient | | 749.78 | | | 72 |  |  |
| (2) | Benin | 2015 ^c^ |  | √ |  |  |  | √ | √ |  | √ |  | √ |  |  |  |  |  |  |  |  |  |  | √ | 2 | | Patient | | 574.30 - 2243 ^g^ | | | 39 |  |  |
| (2) | Burkina | 2015 ^c^ |  | √ |  |  |  | √ | √ |  | √ |  | √ |  |  |  |  |  |  |  |  |  |  | √ | 2 | | Patient | | 628.27 - 2409.32 ^g^ | | | 39 |  |  |
| (2) | Guinea | 2015 ^c^ |  | √ |  |  |  | √ | √ |  | √ |  | √ |  |  |  |  |  |  |  |  |  |  | √ | 2 | | Patient | | 22.25 - 193.04 ^g^ | | | 45 |  |  |
| (2) | Mali | 2015 ^c^ |  | √ |  |  |  | √ | √ |  | √ |  | √ |  |  |  |  |  |  |  |  |  |  | √ | 2 | | Patient | | 375.71 - 2383.15 ^g^ | | | 46 |  |  |
| (3) | Ghana | 2016 | √ |  |  |  |  | √ |  |  |  |  |  |  |  |  |  |  |  |  |  |  | √ |  | 2 | | Societal | | 2185.62 - TTT and 2183.21 - BTT^h^ | | | 27 |  |  |
| (3) | South Africa | 2016 | √ |  |  |  |  | √ |  |  |  |  |  |  |  |  |  |  |  |  |  |  | √ |  | 2 | | Societal | | 1299.63 - TTT and 1287.58 - BTT^h^ | | | 6 |  |  |
| (5) | Seychelles | 2006 ^c^ |  | √ |  |  |  | √ | √ |  |  |  |  |  |  |  |  |  |  |  |  |  | √ |  | 1 & 2 | | Health system ^e^ | | 42.28 | | |  |  |  |
| (8) | Sudan | 2005 |  | √ |  |  |  | √ | √ |  |  |  |  |  |  |  |  |  |  |  |  | √ |  |  | 2 | | Family/patient ^e^ | | 80229.78 ^b^ | | | 76 |  |  |
| (10) | Nigeria | 2013 |  | √ |  |  | √ | √ | √ |  |  |  |  |  |  |  |  |  |  |  |  |  | √ |  | 1 & 2 | | Patient | | 104.98 / month p.p | | | 72 |  |  |
| (12) | Nigeria | 2009-10 |  | √ |  |  | √ | √ | √ |  |  |  |  |  |  |  |  |  |  |  |  |  | √ |  | 2 | | Patient | | 939.91 | | | 72 |  |  |
| (16) | Kenya | 2012 |  | √ |  |  | √ | √ |  |  |  |  |  |  |  |  |  |  |  |  |  |  | √ |  | 2 | | Family/patient ^e^ | | 15.58 - consultation/month ^b,d^ p.p 7.93 - transport/month p.p ^b,d^ | | | 26 |  |  |
| (19) | Benin | 2013-15 |  |  |  |  |  | √ | √ |  | √ |  |  |  |  |  |  |  |  |  |  |  |  | √ | 1 | | Family | | 1345 | | | 39 |  |  |
| (19) | Burkina | 2013-15 |  |  |  |  |  | √ | √ |  | √ |  |  |  |  |  |  |  |  |  |  |  |  | √ | 1 | | Family | | 2807 | | | 39 |  |  |
| (19) | CAR | 2013-15 |  |  |  |  |  | √ | √ |  | √ |  |  |  |  |  |  |  |  |  |  |  |  | √ | 1 | | Family | | 1176 | | | 46 |  |  |
| (19) | Ivory Coast | 2013-15 |  |  |  |  |  | √ | √ |  | √ |  |  |  |  |  |  |  |  |  |  |  |  | √ | 1 | | Family | | 1178 | | | 51 |  |  |
| (19) | Malawi | 2013-15 |  |  |  |  |  | √ | √ |  | √ |  |  |  |  |  |  |  |  |  |  |  |  | √ | 1 | | Family | | 2467 | | | 11 |  |  |
| (19) | Mauritania | 2013-15 |  |  |  |  |  | √ | √ |  | √ |  |  |  |  |  |  |  |  |  |  |  |  | √ | 1 | | Family | | 1927 | | | 44 |  |  |
| (20) | Nigeria | 2015 ^c^ |  | √ |  |  | √ | √ | √ | √ | √ |  |  |  | √ | √ | √ |  |  |  |  | √ |  |  | 2 | | Patient | | 645 / month p.p | | |  |  |  |
| (22) | Ghana | 2009 |  | √ | √ | √ |  | √ | √ |  | √ | √ | √ |  |  |  |  |  |  |  |  | √ |  |  | 1 & 2 | | Health system | | 1090 | | | 72 |  |  |
| (23) | Uganda | 2011 |  |  |  |  | √ | √ | √ |  |  | √ | √ | √ |  |  |  |  |  |  |  |  |  | √ | n.s | | Health system | | 7.24 / visit p.p ^f^ | | | 27 |  |  |
| (24) | Nigeria | 2003-4 |  |  |  |  | √ | √ | √ |  |  |  | √ |  |  |  |  |  |  |  |  |  | √ |  | 1 & 2 | | Health system & patient | | 1143 | | | 72 |  |  |
| (25) | Nigeria | 2011-12 |  | √ |  |  | √ | √ | √ |  |  |  |  |  |  |  |  |  |  |  |  |  |  | √ | 1 & 2 | | Societal | | 616 | | | 72 |  |  |
| (26) | South Africa | 2012 |  | √ |  |  |  | √ | √ |  | √ | √ |  |  |  |  |  |  |  |  |  |  |  |  | 2 | | Government | | 881.35 | | | 6 |  |  |
| **HOSPITALIZATION COSTS** | | | | | | | | | | | | | | | | | | | | | | | | | | | | | | | |  |  |  |
| (6) | Tanzania | 2010 |  | √ | √ | √ |  | √ | √ |  | √ |  |  |  |  | √ |  |  |  |  |  |  | √ |  | n.s | | Patient | | 0.62 and 18.76 | | | 72 |  |  |
| (7) | Nigeria | 2014 |  |  | √ | √ |  | √ | √ |  | √ |  |  |  |  |  | √ |  |  |  |  |  | √ |  | 2 | | Health system ^e^ | | 1180 - 3839.76 | | | 32 |  |  |
| (11) | Ethiopia | 2000-02 |  | √ | √ |  |  | √ | √ |  |  |  |  |  |  |  |  |  |  |  |  |  | √ |  | 1 & 2 | | Health system ^e^ | | 795 ^d^ | | | 34 |  |  |
| (15) | Zimbabwe | 2012-13 | √ |  |  |  |  | √ |  |  | √ |  |  |  |  |  |  |  |  |  |  | √ |  |  | 2 | | Societal | | 2605 | | | 6 |  |  |
| (17) | South Africa | 2009 |  |  | √ | √ |  | √ | √ |  |  |  |  |  |  |  |  |  |  |  |  |  | √ |  | n.s | | Health system ^e^ | | 6871 ^d^ | | | 72 |  |  |
| (18) | Nigeria | 2003-04 |  |  | √ | √ |  | √ | √ |  | √ |  |  |  |  | √ |  |  |  |  |  |  | √ |  | 1 & 2 | | Patient ^e^ | | 2899.5 | | | 6 |  |  |
| (21) | South Africa | 2005 |  |  |  |  |  | √ | √ |  |  | √ | √ | √ |  |  |  |  |  |  |  | √ |  |  | n.s | | Health system & patient ^e^ | | 1813 | | | 27 |  |  |
| (22) | Ghana | 2009 |  | √ | √ | √ |  | √ | √ |  | √ | √ | √ |  |  |  |  |  |  |  |  | √ |  |  | 1 & 2 | | Health system | | 1904 | | | 72 |  |  |
| **COMBINED COSTS** | | | | | | | | | | | | | | | | | | | | | | | | | | | | | | | |  |  |  |
| (4) | Morocco | 2013 ^c^ |  | √ | √ |  |  | √ | √ |  | √ |  |  |  |  |  |  | √ | √ | √ | √ |  |  |  | 1 & 2 | | Societal | | 611.72 to 1960.33 (2628) | | | 58 |  |  |
| (13) | WHO African region | 2005 |  | √ | √ |  | √ | √ | √ | √ | √ |  | √ | √ | √ |  | √ | √ | √ | √ | √ |  |  |  | 1 & 2 | | Societal | | 1377.32 (2958.64) | | |  |  |  |

* Reference index in Appendix 1, Except were indicated all outpatient costs presented are costs incurred per annum per patient, hospitalization costs are admission costs per patient, combined costs are costs per annum per patient, n.s: Not specified, OOP – out of pocket, THE – total health expenditure, p.d: per day.

^a^ Presented costs are average costs except were indicated

^b^ Presented costs are median costs

^c^ Year of costing was unclear in study therefore the year of publication was taken as proxy

^d^ OANDA FX currency converter was used to convert currency to USD

^e^ Perspective is based on reviewer interpretation

^f^ The study provided costs incurred at different types of health facilities – costs presented are those incurred at a hospital

^g^ The study provided the minimum and maximum costs of care in the private and public health care sector of reach country. The results presented here are the maximum and minimum costs reported

^h^ Cost effectiveness study which provided the costs of two treatment strategies (TTT and BTT) in five LMIC. Results presented in this review are for the 2 African countries studied.

^i^ Note that categories are those visible to the reviewer or explicitly stated in the manuscript - these might not be exhaustive
