# Supplementary material for: Cost of diabetes mellitus in Africa: a systematic review of existing literature
Source: Global Health. 2018 Jan 16;14:3. doi: 10.1186/s12992-017-0318-5 (PMC5771003; doi:10.1186/s12992-017-0318-5)
Supplement: Supplementary file 5 — Cost ratio for individuals with and without diabetes mellitus complications (DOCX 21 kb) [file 12992_2017_318_MOESM5_ESM.docx]

Additional file 5: Cost ratio for individuals with and without diabetes mellitus complications

| **Ref** | **Complication** | **Country** | **Diabetes type** | **Complicated diabetes** | **Uncomplicated diabetes** | **Cost ratio** | **Average** |
| --- | --- | --- | --- | --- | --- | --- | --- |
| 2 | Diabetic foot | Benin | 2 | 1880 | 574.3 | 3.27 | 3.78 |
| 2 | Diabetic foot | Burkina Faso | 2 | 2588.83 | 628.27 | 4.12 |  |
| 2 | Diabetic foot | Guinea | 2 | 93.08 | 22.25 | 4.18 |  |
| 2 | Diabetic foot | Mali | 2 | 1327.32 | 375.71 | 3.53 |  |
| 25 | Hypertension | Nigeria | 1 | 1491.87 | 1307.83 | 1.14 | 1.80 |
| 25 | Hypertension | Nigeria | 2 | 779.31 | 595.28 | 1.31 |  |
| 2 | Hypertension | Benin | 2 | 934.58 | 574.3 | 1.63 |  |
| 2 | Hypertension | Burkina Faso | 2 | 1057.41 | 628.27 | 1.68 |  |
| 2 | Hypertension | Guinea | 2 | 64.82 | 22.25 | 2.91 |  |
| 2 | Hypertension | Mali | 2 | 803.52 | 375.71 | 2.14 |  |
| 25 | Hypertension and Cholesterol | Nigeria | 1 | 2303.77 | 1307.83 | 1.76 | 2.24 |
| 25 | Hypertension and Cholesterol | Nigeria | 2 | 1623.8 | 595.28 | 2.73 |  |
| 2 | Infected diabetic foot | Benin | 2 | 1972.11 | 574.3 | 3.43 | 4.27 |
| 2 | Infected diabetic foot | Burkina Faso | 2 | 2044.7 | 628.27 | 3.25 |  |
| 2 | Infected diabetic foot | Guinea | 2 | 117.8 | 22.25 | 5.29 |  |
| 2 | Infected diabetic foot | Mali | 2 | 1914.2 | 375.71 | 5.09 |  |
| 2 | Ischemic heart disease | Benin | 2 | 1449.28 | 574.3 | 2.52 | 2.61 |
| 2 | Ischemic heart disease | Burkina Faso | 2 | 1391.18 | 628.27 | 2.21 |  |
| 2 | Ischemic heart disease | Guinea | 2 | 55.28 | 22.25 | 2.48 |  |
| 2 | Ischemic heart disease | Mali | 2 | 1209.4 | 375.71 | 3.22 |  |
| 2 | Keto acidosis | Benin | 2 | 1099.83 | 574.3 | 1.92 | 1.79 |
| 2 | Keto acidosis | Burkina Faso | 2 | 1085.46 | 628.27 | 1.73 |  |
| 2 | Keto acidosis | Guinea | 2 | 39.21 | 22.25 | 1.76 |  |
| 2 | Keto acidosis | Mali | 2 | 666.4 | 375.71 | 1.77 |  |
| 2 | Nephropathy | Benin | 2 | 2302.6 | 574.3 | 4.01 | 4.38 |
| 2 | Nephropathy | Burkina Faso | 2 | 2196.15 | 628.27 | 3.50 |  |
| 2 | Nephropathy | Guinea | 2 | 142.71 | 22.25 | 6.41 |  |
| 2 | Nephropathy | Mali | 2 | 1346.52 | 375.71 | 3.58 |  |
| 2 | Retinopathy | Benin | 2 | 866.86 | 574.3 | 1.51 | 1.41 |
| 2 | Retinopathy | Burkina Faso | 2 | 858.27 | 628.27 | 1.37 |  |
| 2 | Retinopathy | Guinea | 2 | 33.03 | 22.25 | 1.48 |  |
| 2 | Retinopathy | Mali | 2 | 485.41 | 375.71 | 1.29 |  |
| 2 | Stroke | Benin | 2 | 631.18 | 574.3 | 1.10 | 1.08 |
| 2 | Stroke | Burkina Faso | 2 | 675.96 | 628.27 | 1.08 |  |
| 2 | Stroke | Guinea | 2 | 23.84 | 22.25 | 1.07 |  |
| 2 | Stroke | Mali | 2 | 403.13 | 375.71 | 1.07 |  |
| 2 | Acute Stroke | Benin | 2 | 1725.58 | 574.3 | 3.00 | 15.72 |
| 2 | Acute Stroke | Burkina Faso | 2 | 1615.56 | 628.27 | 2.57 |  |
| 2 | Acute Stroke | Guinea | 2 | 1247.79 | 22.25 | 56.08 |  |
| 2 | Acute Stroke | Mali | 2 | 455 | 375.71 | 1.21 |  |
